# Personal characteristics related to the risk of adolescent internet addiction: a survey in Shanghai, China

**DOI:** 10.1186/1471-2458-12-1106

**Published:** 2012-12-22

**Authors:** Jian Xu, Li-xiao Shen, Chong-huai Yan, Howard Hu, Fang Yang, Lu Wang, Sudha Rani Kotha, Li-na Zhang, Xiang-peng Liao, Jun Zhang, Feng-xiu Ouyang, Jin-song Zhang, Xiao-ming Shen

**Affiliations:** 1 Xinhua Hospital, MOE-Shanghai Key Laboratory of Children’s Environmental Health, Shanghai Institute for Pediatric Research, Shanghai Jiao Tong University School of Medicine, Shanghai, China; 2 Environmental Health Department, School of Public Health, University of Michigan, Ann Arbor, MI, USA; 3 Center for Global Health, University of Michigan, Ann Arbor, MI, USA; 4 Biostatistics Department, School of Public Health, University of Michigan, Ann Arbor, MI, USA; 5 Biostatistics Department, Shanghai Jiao Tong University School of Medicine, Shanghai, China; 6 DALLA LANA School of Public Health, University of Toronto, Ontario, Canada; 7 Center for Global Health, University of Toronto, Ontario, Canada

**Keywords:** Adolescent, Internet addiction, Risk factors, China

## Abstract

**Background:**

Paralleling the rapid growth in computers and internet connections, adolescent internet addiction (AIA) is becoming an increasingly serious problem, especially in developing countries. This study aims to explore the prevalence of AIA and associated symptoms in a large population-based sample in Shanghai and identify potential predictors related to personal characteristics.

**Methods:**

In 2007, 5,122 adolescents were randomly chosen from 16 high schools of different school types (junior, senior key, senior ordinary and senior vocational) in Shanghai with stratified-random sampling. Each student completed a self-administered and anonymous questionnaire that included DRM 52 Scale of Internet-use. The DRM 52 Scale was adapted for use in Shanghai from Young’s Internet Addiction Scale and contained 7 subscales related to psychological symptoms of AIA. Multiple linear regression and logistic regression were both used to analyze the data.

**Results:**

Of the 5,122 students, 449 (8.8%) were identified as internet addicts. Although adolescents who had bad (*vs*. good) academic achievement had lower levels of internet-use (*p* < 0.0001), they were more likely to develop AIA (odds ratio 4.79, 95% CI: 2.51-9.73, *p* < 0.0001) and have psychological symptoms in 6 of the 7 subscales (not in Time-consuming subscale). The likelihood of AIA was higher among those adolescents who were male, senior high school students, or had monthly spending >100 RMB (all *p*-values <0.05). Adolescents tended to develop AIA and show symptoms in all subscales when they spent more hours online weekly (however, more internet addicts overused internet on weekends than on weekdays, *p *< 0.0001) or when they used the internet mainly for playing games or real-time chatting.

**Conclusions:**

This study provides evidence that adolescent personal factors play key roles in inducing AIA. Adolescents having aforementioned personal characteristics and online behaviors are at high-risk of developing AIA that may compound different psychological symptoms associated with AIA. Spending excessive time online is not in itself a defining symptom of AIA. More attention is needed on adolescent excessive weekend internet-use in prevention of potential internet addicts.

## Background

Paralleling the rapid growth in internet access is a rise in internet addiction, especially among adolescents, gaining increased attention from the popular media, government authorities, and researchers [1]. Internet addiction is characterized by a maladaptive pattern of internet use leading to clinically significant impairment or distress [2].

Internet addiction may interfere with people’s daily lives, and had short and long-term effects on their social, psychological and physical well-being. According to previous studies, internet addiction was associated with obsessive-compulsive and depressive disorders, attention-deficit hyperactivity disorder, hostility/ aggressive behaviors, impaired executive control ability, and multiple structural changes in the brain [3-6].

However, there is currently no standardized definition or diagnosis criteria for internet addiction. Based on empirical diagnostic interviews and epidemiological studies, Young and Ko et al. [2,4,6] proposed their diagnostic criteria for Internet addiction in which withdrawal, poor planning abilities, tolerance, preoccupation, impairment of control, and excessive online time were defined as core symptoms of Internet addiction. Until now, internet addiction is a proposed but unproven disorder, and the upcoming inclusion of Internet addiction in the DSM-V as a disorder in need of further study compels further investigation.

Adolescents (also called teen-ages) usually have poorer self-control, worse self-regulation, and poorer cognition than college-age populations or adults, but they have the same desire for independence as college-age populations or adults do. On the other hand, nowadays, computer use by adolescents is encouraged, and in some courses is required. The use of the internet is logical and common even outside the classroom. Most of the high school libraries, families, and internet café have internet access, and adolescents are easy to have access to the internet. In addition, parents exercise less control over adolescents than younger children in elemental schools or kindergartens. Therefore, adolescents are considered as the most vulnerable group to the temptations of the internet [7,8].

Current US data suggest that 93% of young people between the ages of 12 and 29 years have used the internet at least once [9]. According to the statistical report of China Internet Network Information Center, over 500 million people in China had access to the Internet as of September 2011. Of those, approximately 33% were teenagers below 18 years of age and 60% were between 10–29 years of age [8,10].

In addition to genetic factors such as presence of the SS-5HTTLPR gene, family and environmental factors, previous studies have shown the possibility that personal factors may play a key role in internet use and the development of adolescent internet addiction (AIA) [2]. Adolescent personality traits that correlated positively with internet addiction included high harm-avoidance, reward dependence, low self-esteem, and low cooperativeness [2]. Yet previous studies may have limitations. First, some studies that addressed the inherent personality of adolescents susceptible to AIA by using psychological measurements had shown inconsistent results [11,12]. Secondly, those studies that were conducted online or on relatively small sample sizes, raising the question of their representativeness of the general population [13]. Thirdly, some studies found that drinking and smoking behaviors were potential risk factors for AIA, but these two risk factors are surely insufficient to characterize all the risk factors of AIA [2,7]. Finally, many studies were performed among college students who were typically 18 years of age, older than the adolescent range of 12–17 years of age.

Therefore, we performed a large cross-sectional survey in 16 high schools of Shanghai city, of which, the aim was to investigate the prevalence of AIA among high school students and to assess whether the students’ personal characteristics had potential impacts on the risk of AIA.

## Methods

### Study design and population

Study participants were junior high school students and senior high school students in Shanghai, recruited from October to November 2007. A stratified cluster random sampling design was applied to target recruitment. The 19 administrative districts of Shanghai are designated either as urban or suburban areas, depending on their geographic features and levels of economic development. Six administrative districts were selected based on their geographical location, population density, and socioeconomic characteristics, and within those districts, 16 schools were selected in an equal ratio (1:1) of urban to suburban areas. In each area, 2 junior high schools, 2 ordinary senior high schools, 2 key senior high schools, and 2 vocational senior high schools were randomly selected, maintaining the same 1:1 distribution of each type of school in the area. All students in each of the selected schools were invited to participate in this research. A total of 5,135 adolescents from 1^st^ year of junior high school to 3^rd^ year of senior high school participated in the study. Of these, 5,122 (99.8%) returned completed questionnaires.

School approval and parental informed consent for adolescent participation were obtained for all students who participated. The study was approved by the medical ethical committee of Xinhua Hospital, affiliated to Shanghai Jiao Tong University School of Medicine.

### Data collection and measures of AIA

All questionnaires were distributed to participants in classroom settings at a predetermined time and were collected on-site after 30 minutes. Questionnaires were anonymous and self-administered. Teachers left the classrooms during the 30-minute period to avoid any possible information bias.

The questionnaire contained 3 parts: 1) adolescent personal demographic information including gender, age, grade and the type of school, 2) school performances (self-rated academic achievement) and family factors that could potentially influence adolescent internet use, and 3) adolescent internet-use behaviors [see Additional file 1].

The scientific literature contains a considerable diversity of opinions on the diagnostic criteria for internet addiction [2,14,15]. We utilized the DRM 52 Scale of Internet-use in Adolescents (DRM 52 Scale), developed from the Young’s Internet Addiction Scale [15], validated in 2005, and adapted for use in Shanghai to investigate the prevalence of AIA since 2005 [16]. The adjusted scale includes all contents of Young’s scale but uses both direct and indirect questions to get as accurate information as possible. The additional file shows this questionnaire and DRM 52 Scale in more detail [see Additional file 1.

All answers of 52 items were coded on a 5-point scale from 1 (completely disagree) to 5 (fully agree). The total score on the DRM 52 Scale ranged from 0 to 260. A score of 0 meant that a student never used the internet. Internet addiction was defined if the total score was over 163 [16], and scores still higher indicated increasing severity of internet addiction.

The 52 items were grouped into seven subscales: Tolerance (whether the same amount of internet usage elicits a response of less satisfaction for the user), Withdrawal reaction (whether the user feels unwell when not online), Planning (whether the user poorly follows his/her plan of internet usage), Lack-of-control (whether the user is able to control his/her on-line use), Time-consuming (whether the user spends too much time online), Socialization (level of interference in interpersonal relationships) and Negative-life-consequences (negative consequences on their life including health status).

An internal comparison of the psychometric scores on the DRM 52 Scale demonstrated good internal consistency of the overall questionnaire and seven subscales (Cronbach alpha was around 0.7), and excellent test–retest reliability (intraclass correlation coefficient was 0.75) [16].

### Statistical analysis

Statistical descriptions were used to describe students’ demographic characteristics and general information of adolescent internet use. Differences in total scores and differences in the rates of AIA among different levels within the same categories were analyzed by one-way ANOVA and Chi-square test, respectively. Logistic regression analyses were performed to analyze potential risk factors for AIA, including those covariates that had significant (*p *< 0.05) bivariate associations with AIA and using “1” for adolescents with total scores of DRM 52 Scale >163 and “0” for adolescents with total scores ≤163. To evaluate the association between presence of related factors influencing adolescent internet usage and total scores of DRM 52 Scale or each subscale, multiple linear regression analyses were conducted with stepwise models to examine the association between adolescent personal factors and symptoms of seven subscales. In order to reduce the number of potential covariates with low explanatory power, bivariate correlation analyses were performed before linear regressions. Because of the high correlation between ‘total hours spent online weekly’ and ‘total hours spent online on weekends’ (Pearson correlation r=0.83), we chose ‘total hours spent online weekly’ to represent the general value of total hours spent online. We merged 1^st^ to 3^rd^ of junior high school years as the variable ‘junior high school students’ and merged 1^st^ to 3^rd^ of senior high school years as the variable ‘senior high school students’.

All analyses were performed using SAS (version 9.2; SAS Institute Inc., Cary, NC). In presenting results, statistical significance was set at *p *< 0.05 (two tailed).

## Results

### Personal characteristics of adolescent internet addicts

The average age of participants was 15.9 years, with a range of 11.3 to 20.4 years (no repeater students), of whom 49.6% were boys and 50.4% were girls. For all 5,122 subjects, the average level of total scores and the average levels of subscale scores on the DRM 52 scale were within the normal range. Of all 5,122 subjects, 4,383 (85.5%) reported using the internet rarely or moderately, and 449 participants (8.8%) met the criteria for internet addiction (see Table 1).

**Table 1:** Personal characteristics and characteristics of online behaviors of the study population of 5,122 Shanghai adolescents

**Variables**	**N**	**Percentage (%)**	**Internet use %(N**_**using internet**_**/ N**_**whole**_**)**^**c**^	**Total Score**^**a**^**(mean ± SD)**	**Internet addiction**^**b**^**%(N**_**internet addiction**_**/ N**_**whole**_**)**^**d**^
**Gender** (**missing n**=**8**)					
Female (ref.)	2580	50.4%	95.1% (2453 / 2580)	117.0 ± 38.1	6.4% (164 / 2580)
Male	2534	49.6%	92.6% (2347 / 2534)**^e^	122.4 ± 43.5***^e^	11.3% (285 / 2534)***^e^
**Grade** (**missing n**=**1**)					
Junior high school students (ref.)		1915	37.4%	92.6% (1774 / 1915)	110.3 ± 42.4	7.1% (135 / 1915)
Senior high school students		3206	62.6%	94.6% (3033 / 3206)**	125.3 ± 39.0***	9.8% (315 / 3206)**
**School type** (**missing n**=**2**)						
Junior high school (ref.)		1907	37.2%	92.7% (1768 / 1907)	110.4 ± 42.3	7.1% (135 / 1907)
Key senior high school		1096	21.4%	94.5% (1036 / 1096)	120.2 ± 37.7***	6.1% (67 / 1096)
Ordinary senior high school		1102	21.5%	93.3% (1028 / 1102)	122.9 ± 40.9***	9.4% (104 / 1102)*
Vocational senior high school		1015	19.8%	96.0% (974 / 1015)*	133.1 ± 37.3***	14.2% (144 / 1015)***
**Adolescent monthly money spending** (**missing n=9**)						
< 100 RMB (ref.)		3028	59.1%	92.6% (2805 / 3028)	112.2 ± 41.1	5.6% (168 / 3028)
100~300 RMB		1373	26.8%	95.7% (1314 / 1373)***	128.9 ± 38.0***	12.5% (172 / 1373)***
≥300 RMB		712	13.9%	95.7% (681 / 712)***	133.5 ± 38.3***	15.3% (109 / 712)***
**Residential or commuter students** (**missing n=4**)						
Commuter students (ref.)		4667	91.1%	93.7% (4372 / 4667)	119.2 ± 41.3	8.7% (407 / 4667)
Residential students		451	8.8%	96.0% (433 / 451)*	125.2 ± 35.6**	9.5% (43 / 451)
**Academic achievement** (**missing n=16**)						
Very good (ref.)		324	6.3%	92.3% (299 / 324)	113.1 ± 42.2	4.0% (13 / 324)
Relatively good		1386	27.1%	94.4% (1308 / 1386)	112.9 ± 38.3	5.6% (78 / 1386)
General		2835	55.4%	94.6% (2682 / 2835)	122.3 ± 39.4***	8.9% (253 / 2835)***
Relatively &very bad		561	11.0%	89.7% (503 / 561)	126.8 ± 50.7***	18.9% (106 / 561)***
**Main purpose of using internet** (**missing n=15**)						
Academic learning (ref.)		720	14.1%	100%	109.3 ± 26.8	1.4% (10 / 720)
Only browsing news or emails		881	17.2%	100%	117.3 ± 25.7***	2.5% (22 / 881)
Playing games		1003	19.6%	100%	140.7 ± 28.6***	20.1% (202 / 1003)***
Real-time chatting		667	13.0%	100%	132.5 ± 24.6***	8.1% (54 / 667)***
Wandering aimlessly online		1546	30.2%	100%	128.9 ± 29.2***	10.2% (158 / 1546)***
**Place of using internet** (**missing n=24**)						
School (ref.)		314	6.1%	100%	115.2 ± 29.4	4.5% (14 / 314)
Residences of classmates or relatives		705	13.8%	100%	120.7 ± 27.6*	5.4% (38 / 705)
Home		3586	70.0%	100%	128.5 ± 29.4***	10.3% (369 / 3586)**
Internet bar		203	4.0%	100%	137.6 ± 25.9***	14.3% (29 / 203)***
**Total hours online for a whole week** (**missing n=16**)						
<7 hours (ref.)		2812	54.9%	100%	117.7 ± 27.7	4.1% (114 / 2812)
7~14 hours		970	18.9%	100%	133.2 ± 26.6***	11.8% (114 / 970)***
14~21 hours		414	8.1%	100%	140.9 ± 24.1***	15.5% (64 / 414)***
21~28 hours		242	4.7%	100%	145.8 ± 23.9***	22.3% (54 / 242)***
>28 hours		378	7.4%	100%	151.1 ± 26.9***	27.3% (103 / 378)***
**Hours online per weekday** (**missing n=21**)						
≤2 hours (ref.)		3390	70.5%	100%	121.9 ± 29.1	6.9%(235/3390)
2~4 hours		756	15.7%	100%	134.3 ± 24.8***	10.6%(80/756)**
4~6 hours		357	7.4%	100%	139.5 ± 26.9***	17.1%(61/357)***
6~8 hours		113	2.3%	100%	143.0 ± 28.0***	23.0%(26/113)***
≥8 hours		195	4.0%	100%	151.4 ± 26.7***	24.2%(47/195)***
**Hours online on each Saturday**/**Sunday** (**missing n=17**)						
≤2 hours (ref.)		1835	35.8%	100%	114.5 ± 28.1	3.4% (63 / 1835)
2~4 hours		1457	28.5%	100%	125.6 ± 25.8***	6.3% (92 / 1457)***
4~6 hours		668	13.0%	100%	136.5 ± 25.2***	12.0% (80 / 668)***
6~8 hours		351	6.9%	100%	143.8 ± 24.5***	20.2% (71 / 351)***
≥8 hours		504	9.8%	100%	150.7 ± 27.5***	28.4% (143 / 504)***

^**a**^ total score: total score of DRM-52 scale.

^b^ internet addiction: defined when the total score of DRM-52 scale ≥163.

^**c**^ %(N_internet use_ /N _whole_): the rates of internet use was calculated as the ratio of the numbers of internet-use adolescents to the numbers of whole adolescent samples in that group.

^d^ %(N_internet addiction_ / N _whole_): the rates of AIA was calculated as the ratio of the numbers of internet-addicted adolescents to the numbers of whole adolescent samples in that group.

^e^*p* values for the comparison of total scores were calculated by one-way ANOVA, *p* values for the comparison of the rates of AIA were calculated by chi-square.

*p-values<0.05; **p-values<0.01; ***p-values<0.001 (compared with reference groups).

As shown in Table 1, total scores of DRM 52 scale were higher for males than for females (*p *= 0.00), and male students comprised 63.3% (285/449) of all internet addicts. Senior high school students showed higher total scores and a higher prevalence rate of AIA than did junior high school students (both *p *< 0.01), among whom the 2^nd^ year senior high school students had the highest total scores and highest rate of internet addiction (127.3±40.3 and 11.7%, respectively). Of all 5,122 subjects, monthly spending greater than 300 RMB were reported by 712 students, and their total scores and their rate of AIA were relatively high (133.5±38.3 and 15.3%, respectively). The 561 students who showed bad and relatively bad academic achievement had higher total scores (126.8±50.7) and higher rate of AIA (18.9%) than those with good achievement (see Table 1).

**Figure 1:**
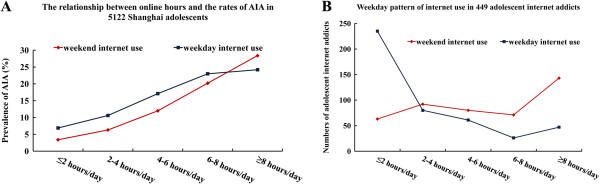
**Online hours and adolescent internet addiction(AIA).** (**A**) The relationship between the online hours per weekend or per weekday and the rates of AIA in 5,122 Shanghai adolescents. Both lines indicated that more hours online on weekdays or weekends were associated with higher rates of AIA. (**B**) Weekday-pattern of internet-use in 449 adolescent internet addicts. Both lines indicated that more internet addicts overused internet on weekends than on weekdays.

In addition, 23.2% of addicted internet users and 8.3% of non-addicted users spent 28 hours or more online weekly. More hours spent online (on weekdays or weekends) were associated with higher rates of AIA (see Table 1 and Figure 1A). Of all 449 addicted internet users, 31.8% spent 8 hours or more online on each weekend (Saturday or Sunday), while only 10.5% did it per weekday. This percentage difference was statistically significant (31.8% *vs* 10.5%, *x*^2^=61.5, *p *< 0.0001, see Figure 1B). Other  demographic and personal characteristics and the distribution of subjects were shown in Table 1.

### Logistic regression analyses for AIA

In multivariate logistic regression analyses, male gender, high monthly spending levels, poor academic achievement, more hours spent online weekly, and games and real-time chatting as the main purposes of internet use were all related to AIA (Table 2). Adolescents whose monthly spending levels ≥300 RMB or 100 to 300 RMB were more likely to develop AIA than adolescents who spent < 100 RMB per month. (odds ratio [OR], 1.51, *p* < 0.01; OR, 1.66, *p* < 0.0001, respectively). Risk for AIA was posed by poor or relatively poor academic achievement (relative to very good achievements, OR, 4.79, *p* < 0.001) and by male gender (relative to females, OR 1.29, *p*=0.036).

**Table 2:** **Impacts of adolescent personal factors on adolescent internet addiction by logistic regression analysis**^**a,b**^

**Risk Factors**	**Coefficient** (**Standard Error**)	**Odds Ratio**	**95%Confidence Interval**	***p-*value**
**Gender**				
Female (ref.)	1.0	1.0		
Male	0.26(0.12)	1.29	1.02-1.64	0.0361
**Adolescent monthly money spending levels** (**RMB **/ **month**)				
<100 (ref.)	1.0	1.0		
≥300	0.41(0.16)	1.51	1.11-2.05	0.0092
100~299	0.51(0.13)	1.66	1.29-2.14	<0.0001
**Academic achievements**				
Very good (ref.)	1.0	1.0		
Very & relatively bad	1.57(0.33)	4.79	2.51-9.13	<0.0001
General	0.87(0.31)	2.38	1.29-4.41	0.0057
Relative good	0.52(0.33)	1.68	0.88-3.20	0.1186
**Total hours online for a whole week** (**hours** /**month**)				
<7 (ref.)	1.0	1.0		
>28	1.45(0.17)	4.28	3.06-5.99	<0.0001
21 ~28	1.23(0.21)	3.41	2.26-5.15	<0.0001
14 ~21	0.96(0.19)	2.61	1.81-3.77	<0.0001
7~14	0.89(0.15)	2.44	1.81-3.29	<0.0001
**Main Purpose of using internet**				
Academic learning (ref.)	1.0	1.0		
Playing game	1.94(0.34)	6.98	3.59-13.58	<.0.0001
Real-time chatting	0.97(0.36)	2.64	1.30-5.38	0.0073
Browsing news or e-mails only	0.17(0.40)	1.19	0.55-2.60	0.6625

^a^ This logistic regression model was fit to model the possibility of adolescent having internet addiction, internet addiction was defined as total score ≥ 163.

^b^ Adolescent age, gender, grade, school types, adolescent academic achievement, adolescent monthly spending levels, internet-use time, and the main purposes and places of adolescent internet use were adjusted in the models.

Adolescents who spent >28 hours, 21 to approximately 28 hours, 14 to 21 hours or 7 to 14 hours online per week were more likely to develop AIA than adolescents who spent <7 hours. (OR: 4.28, *p* < 0.001; OR: 3.41, *p* < 0.001; OR: 2.61, *p* < 0.001; OR: 2.44, *p* < 0.001, respectively). AIA was associated with the purpose of going online of playing games (playing games *vs* academic learning, OR 6.98, *p *< 0.001), and the purpose of going online of real-time chatting (real-time chatting *vs* academic learning, OR 2.64, 95% CI 1.30-5.38, *p*=0.007) (Table 2).

### Multivariate linear regression analyses for AIA

In the multivariate linear regression model, personal characteristics relating to AIA included male gender (*p* <0.001), senior high school students (*p* < 0.001), having poor academic achievements (*p* < 0.001) and high monthly spending (*p* < 0.001) (see Table 3).

**Table 3:** **Impacts of personal factors on the symptom development of AIA by linear regression analyses**^**a,b,c,d**^

**Risk factors**	**Total score** (**Adjusted R**^**2**^=**0**.**31**)	**Seven subscales**						
		**Lack**-**of**- **control**	**Socialization**	**Planning**	**Negative life consequences**	**Time consuming**	**Tolerance**	**Withdrawal**
**Gender**								
*G*irls (ref.)	1.0	1.0	1.0	1.0	1.0	1.0	1.0	1.0
Boys	3.9 (0.8)***	0.2(0.1)	0.6(0.2)***	0.0(0.1)	0.3 (0.1)*	1.0(0.1)***	0.5 (0.1)***	1.5(0.3)***
**Grade**								
Junior high school students (ref.)	1.0	1.0	1.0	1.0	1.0	1.0	1.0	1.0
Senior high school students	8.2 (0.9)***	0.7(0.1)***	2.5(2.3)	1.7(1.6)	1.7 (0.2)***	2.4(1.6)	1.0 (0.1)***	2.0(0.3)***
**Monthly money spending **(**RMB**/**month**)								
<100 (ref.)	1.0	1.0	1.0	1.0	1.0	1.0	1.0	1.0
≥300	6.1(1.2)***	0.5(0.2)**	1.0(0.3)***	0.9(0.2)**	0.8(0.2)***	1.0(0.2)***	0.4(0.2)***	1.7(0.5)**
100~299	6.4 (1.0)***	0.5(0.1)***	0.9(0.2)***	0.7(0.1)***	0.9 (0.2)***	0.9(0.1)***	0.5(0.1)***	1.9(0.4)***
**Academic achievements**								
Very good (ref.)	1.0	1.0	1.0	1.0	1.0	1.0	1.0	1.0
Relatively & very bad	12.6(2.0)***	1.3(0.3)***	4.0(0.4)***	1.6(0.3)***	1.3(0.3)***	0.2(0.3)	1.4(0.3)***	2.7(0.7)***
General	4.8(1.6)**	0.8(0.2)***	1.8(0.3)***	0.8(0.2)***	0.4(0.3)	0.0(0.2)	0.5(0.2)*	0.9(0.5)
Relatively good	−1.3(1.6)	0.1(0.2)	0.4(0.3)	0.2(0.2)	−0.4(0.3)	−0.4(0.2)	−0.1(0.2)	−1.1(0.6)
**Purposes to use internet**								
Academic learning (ref.)	1.0	1.0	1.0	1.0	1.0	1.0	1.0	1.0
Games	20.7(1.4)***	2.0(0.2)***	3.6(0.3)***	2.6(0.2)***	3.0(0.3)***	2.0(0.2)***	1.5(0.2)***	6.5(0.5)***
Real-time chatting	13.6(1.5)***	1.4(0.2)***	2.2(0.3)***	1.8(0.2)***	1.9(0.3)***	1.4(0.2)***	1.0 (0.2)***	4.3(0.6)***
Browsing news or e-mails	4.0 (1.4)**	0.4(0.2)	0.4(0.3)	0.7 (0.2)***	0.7 (0.2)**	0.5 (0.2)*	0.1(0.2)	1.4 (0.5)**
**Total hours spent on internet for a whole week** (**hours**/**week**)								
<7	1.0	1.0	1.0	1.0	1.0	1.0	1.0	1.0
>28	19.9(1.5)***	0.7(0.2)***	3.4(0.3)***	2.4(0.2)***	2.2(0.3)***	3.2(0.2)***	3.3(0.2)***	5.6(0.6)***
21 ~28	17.1(1.8)***	0.9(0.3)***	2.6(0.4)***	2.1(0.3)***	1.9(0.3)***	2.1(0.3)***	2.7(0.2)***	5.5(0.7)***
14 ~21	14.5(1.4)***	0.8(0.2)***	2.6(0.3)***	1.8(0.2)***	1.6(0.3)***	1.7(0.2)***	2.4(0.2)***	4.0(0.5)***
7~14	10.2(1.0)***	0.7(0.1)***	1.7(0.2)***	1.2(0.2)***	1.2(0.2)***	1.2(0.2)***	1.5(0.1)***	2.8(0.4)***
**School type**								
Junior high school	1.0	1.0	1.0	1.0	1.0	1.0	1.0	1.0
Vocational senior high school	7.1(11.4)	2.2(1.6)	2.1(0.2)***	1.7(0.2)***	0.6(2.0)	2.2(1.6)	0.2(1.4)	3.9(3.9)
Ordinary senior high school	1.9(11.3)	1.4(1.5)	1.2(0.2)***	1.5(0.2)***	0.2(1.9)	2.8(1.6)	0.4(1.4)	3.0(3.9)
Key senior high school	2.9(11.3)	1.6(1.6)	0.8(0.3)**	0.7(0.2)***	0.2(1.9)	2.6(1.6)	0.3(1.4)	3.6(3.9)

^a^ Results are reported as Coefficient Estimate (SE).

^b^ AIA: adolescent internet addiction.

^c *^p-values<0.05; ^**^p-values<0.01; ^***^p values<0.001.

^d^ Adolescent age, gender, grade, school types, adolescent academic achievement, adolescent monthly spending levels, internet-use time, and the main purposes and places of adolescent internet use were adjusted in the models.

Detailed effects of these personal factors were further analyzed using the 7 subscales of DRM 52 Scale. Compared to adolescents with low monthly spending (<100 RMB/month), adolescents with high monthly spending (≥ 300 RMB/month or 100~300 RMB/ month) tended to develop AIA with significant symptoms in all subscales (*p* values for all 7 subscales < 0.001). Adolescents with relatively bad and very bad academic achievement had significant symptoms in 6 subscales but not in Time-consuming Subscale (*p* values for Time-consuming Subscale > 0.05, *p* values for all other 6 subscales < 0.001) (Table 3).

The total score of DRM 52 Scale was not significantly associated with school type (*p *> 0.05), yet compared with junior high school students, senior high school students of different school types had a significantly higher likelihood for symptoms of the two subscales (Socialization and Planning subscales, *p* < 0.01), and vocational high school students had the highest coefficient estimates (2.1 for Socialization subscale, 1.7 for Planning subscale), showing the highest risk for developing symptoms of these 2 subscales (Table 3).

Adolescents who spent many hours online (*p *< 0.001), who used the internet mainly for playing games (*p *< 0.001) or for real-time chatting (*p *< 0.001), tended to develop internet addiction. Both groups of adolescents were susceptible to the symptom development on all 7 subscales (all *p* values < 0.001).

## Discussion

### Prevalence of AIA in Shanghai

In our study, of 5,122 adolescents, 8.8% met the criteria of addiction. Previous epidemiological studies showed that the international prevalence rates of AIA ranged from 0.9% to 38% [2,7]. In China, Cao et al. recently investigated 17,599 students in eight cities of China using Young’s Internet Addiction scale and reported an 8.1% rate of AIA [17]. Wang et al. performed a cross-sectional study in Guangdong adolescents using Young’s scale and found that 12.2% of those surveyed met the criteria for AIA [7]. Using a modified version of Young’s scale on 2,620 high school students in Changsha city in 2007, Cao and Su, reported a 2.4% rate of AIA [18]. King-Wa et al. investigated 208 adolescents in Hong Kong in 2010 using Young’s scale and reported a 6.7% rate of AIA [19]. In other countries, the prevalence of AIA using Young’s scale varied from 1.5% to 10% [2]. Pallanti et al. collected data of 275 Italian high school students using Young’s scale in 2006 and reported a 5.4% rate of AIA [20]. In a cross-sectional study of 866 Greek adolescents in 2007, Kormas et al. reported a 1.5% rate [21].

Therefore, AIA in Shanghai can not be ignored, and specific attention needs to be paid to the prevention and treatment of AIA in Shanghai. Shanghai is the commercial and financial center of mainland China. While the increase in computers and the universal application of the internet have brought great convenience to adolescents for recreational and academic use, adolescents in Shanghai should be appropriately supervised to prevent from developing an addiction to the internet.

### Internet-use characteristics of adolescent internet addicts

According to our study, the internet-use characteristics of adolescent Internet addicts included excessive hours spent online, the purpose of using the internet mainly for playing games or for real-time chatting.

While addiction was associated with more hours online on weekends or on weekdays (Figure 1A), more addicts were found to spend 8 hours or more online per weekend than per weekday (Figure 1B), suggesting that excessive weekend internet-use was a more important predictor for AIA than weekday internet over-use. The fact that adolescents had plenty of leisure time on weekends, lack of supervision, and were relatively mentally immature might explain this “weekend” effect. Previous studies have shown that spending excessive hours online was strongly related to internet addiction [7,22], but no other study had focused on the weekday pattern of AIA, a pattern that may well be important for screening potential adolescent addicts.

AIA was significantly associated with the purpose of going online mainly for e-games. This finding was consistent with previous studies showing that excessive computer/video game playing was predictive of internet addiction [2,6,7,23,24]. Some psychiatrists, however, found AIA an independent disorder belonging to impulse control disorders and had suggested that internet addiction and computer/video game playing should both be considered for inclusion in the forthcoming DSM V [1,2].

Online real-time chatting was found to be a definite risk factor for AIA in our study (although emailing alone was not), which was consistent with several previous studies [24,25]. Because of their thirst for independence and emotional appeal, and their relative lack of social experience, adolescents more easily believe strangers met online than do adults. In China, the pathway for real-time online chatting usually includes the instant messaging services such as MSN and QQ. According to media reports, adolescents, especially those from unhealthy families or with personality defects, were susceptible for violent attacks or sexual harassment from people met and chatted online [26,27].

### Adolescent personal characteristics of AIA

Although associated with a lower level of internet-use, poor academic achievement was found to be a significant risk factor for AIA in our study. It could be surmised that lower internet use among students with poor academic achievement was due to their learning disabilities, although few studies have focused on the association between poor academic learning and AIA, much less examined this possibility. Previous studies showed that adolescents with poor academic achievements usually received less respect from surrounding people, and poor academic achievement might be associated with low self-esteem and with behavioral problems such as sleep disorders, aggressive or depressive symptoms, dropping out of school, antisocial personality disorder, and alcohol abuse [28,29]. Such social and psychological corollaries of poor academic achievement were not part of academic evaluation in our study in that an adolescent's academic score was sometimes the only index available to teachers and schools. Perhaps the frustration faced in real world situations would make these adolescents to go online in a search for feelings of fulfillment and self-satisfaction [30].

In addition, our study found that AIA was significantly associated with high monthly spending. Because of the improved standard of living and the national policy of “one child in one family” in China, most adolescents usually get some monthly pocket money from parents (shown in Table 1). Few studies had focused on this aspect when searching for risk factors of AIA. Other studies had shown an association between adolescent high spending and the onset of behavior problems [31-33]. A survey involving 26,454 adolescents in Spain and a survey of 3,634 undergraduate college students in USA both indicated that high spending was independently associated with adolescent heavy drinking, even after all confounding factors were controlled [31,32]. Another study of adolescents in Portugal showed those with high spending levels tended to be associated with increased smoking behaviors [33], and other studies identified smoking and alcohol consumption as potential risk factors of AIA [2,8]. The high spending closely related to AIA might be due to ‘peer effects’ (peer influences on smoking, drinking, and frequent internet usage) [7] and the high cost of frequent online activity. We suggest, therefore, that adolescent high monthly spending levels might be a clue to AIA risk. Prospective studies on the relationship between adolescent monthly spending and AIA needs to be performed later to confirm our results.

### Psychological symptoms of subscales

Our study showed that adolescent with aforementioned personal characteristics and risky online behaviors had significant symptoms in subscales. At least one previous study showed that some psychiatric disorders such as depression, attention-deficit/hyperactivity disorder, social phobia, and hostility were predictive of the occurrence of internet addiction [4]. Another study found a high obsessive-compulsive dimension in adolescent addicts before they became addicted to the internet [34]. After becoming addicted, these adolescents might develop more psychological symptoms, further impeding any adaptive capabilities to the surrounding environment and increasing the possibility of severe AIA. Our results showed internet addiction to online chatting facilitated the development of symptoms on all 7 subscales, consistent with other studies showing that online communication could lead to a decline in adolescent psychosocial well-being and that instant messaging was related to feelings of depression 6 months later [26].

On the other hand, we found that, although strong positive relationship existed between the total hours spent online weekly and the likelihood of AIA, excessive hours spent online appeared not to be a necessary symptom of AIA. According to previous inclusive diagnostic schemas, AIA should be comprehensively assessed according to manifestations of all subscales [2,4,14,15].

### Limitations

This study had several limitations. The first limitation was that adolescent academic achievement was self-reported by adolescents themselves, and a reporting bias was possible. The adolescent respondents might not have honestly reported their internet use and symptoms of internet addiction, despite the fact that questionnaires were answered anonymously and that teachers were kept away from the classrooms where information was being collected. The second limitation was that cross-sectional design of our study raised the possibility of reverse causality, for example, that AIA may be a risk factor for poorer academic performance (rather than the reverse). Finally, there may be other adolescent personal factors relating to AIA that our study missed or did not consider and that were therefore not included as variable candidates in this study.

## Conclusions

Compared with previous studies, this study pointed to the importance of adolescent personal factors in inducing AIA, offered new and detailed associated information, and thus contributed to the literature on the risk of AIA. Our study also confirmed that excessive online time alone was not a defining symptom of AIA, and adolescent excessive weekend internet-use needed more attention for prevention and screening potential internet addicts.

The information will be useful for health care professionals and youth advocates when counseling parents and adolescents on strategies for preventing AIA. A better understanding of AIA requires further analyses to detail risk factors in the adolescent's family and social environment that are as yet uncharted.

## Abbreviations

AIA: Adolescent internet addiction; OR: Odds ratio.

## Competing interests

The authors declare that they have no competing interests.

## Authors’ contributions

JX, Lx-S, Ch-Y, Xp-L participated in subjects' recruitment and the data collection. JX, HH, FY, LW, RK and Ln-Z performed statistical analyses. JX, HH, JZ and Js-Z interpreted the findings. JX wrote the manuscript. HH and RK revised the manuscript. Ch-Y and Xm-S designed the study. Ch-Y obtained funding. All authors read and approved the submitted manuscript.

## Pre-publication history

The pre-publication history for this paper can be accessed here:

http://www.biomedcentral.com/1471-2458/12/1106/prepub

## Supplementary Material

Additional file 1 **Questionnaire and DRM-52 scale. Description of data: In this study, the questionnaire was used to collect adolescent information about individual, familial, and parent-adolescent relationships.** DRM 52 Scale of Internet-use in Adolescents was applied to qualify the prevalence of adolescent internet addiction in Shanghai and to assess the related psychological symptoms of adolescent internet addiciton. We used the Chinese questionnaire and scale in our study. But for peer review, we submitted both the Chinese edition and English edition (translated). Click here for file
