# Supplementary material for: Personal characteristics related to the risk of adolescent internet addiction: a survey in Shanghai, China
Source: BMC Public Health. 2012 Dec 22;12:1106. doi: 10.1186/1471-2458-12-1106 (PMC3563549; doi:10.1186/1471-2458-12-1106)
Supplement: Additional file 1 — Questionnaire and DRM-52 scale. Description of data: In this study, the questionnaire was used to collect adolescent information about individual, familial, and parent-adolescent relationships. DRM 52 Scale of Internet-use in Adolescents was applied to qualify the prevalence of adolescent internet addiction in Shanghai and to assess the related psychological symptoms of adolescent internet addiciton. We used the Chinese questionnaire and scale in our study. But for peer review, we submitted both the Chinese edition and English edition (translated). [file 1471-2458-12-1106-S1.pdf]

Adolescent Internet-use Questionnaire

District________ Name of School________ Grade_____ Date________

Dear students:

This survey is designed to collect your information on your internet-use and to test your tendency to become addicted to the internet. This questionnaire is anonymous. To take this questionnaire, please circle the number next to the selection which best reflects how each statement applies to you, and please answer each one of the questions. You don't have to think about it too hard or too long, there are no correct or wrong answers, and the best of all are those, who come spontaneously. Thank you for participating in this Internet-use survey!

PART 1 General Information

1. Your Gender_______
(1) Male (2) Female

2. Your birthday________________(mm/dd/yy)

3. You are_________
(1) Residential students (2) Commuter students

4. Your father's educational level__________
⑴ Illiteracy/elementary school (2) Junior high school (3) Senior high school (4) University-level or above

5. Your mother's educational level __________
⑴ Illiteracy/elementary school (2) Junior high school (3) Senior high school (4) University-level or above

6. Your father's occupation__________
(1) Workers (manufacturing, production or transport personnel) (2) Peasants (agriculture, forestry, animal husbandry or fishery personnel) (3) Commercial staff (4) Financial staff (5) Teachers (6) researchers (7) Administrators (8) Doctors or nurses (9) Unemployed people (10) Others

7. Your mother's occupation__________
(1) Workers (manufacturing, production or transport personnel) (2) Peasants (agriculture, forestry, animal husbandry or fishery personnel) (3) Commercial staff (4) Financial staff (5) Teachers (6) researchers (7) Administrators (8) Doctors or nurses (9) Unemployed people (10) Others

8. Your average monthly allowance level ___________
(1) Less than 100 RMB (2) 100 to 299 RMB (3) 300 to 599 RMB (4) 600 RMB or more

9. Your average monthly consumption level ____________
(1) Less than 100 RMB (2) 100 to 299 RMB (3) 300 to 599 RMB (4) 600 RMB or more

10. Does your family own the house/apartment you live? _____________
(1) Yes (2) No (rent)

11. Do you have a private bedroom in your home ? ____________
(1) Yes (2) No

12. Does your family have a car or not? ___________________
(1) Yes (2) No

13. What’s your family structure? ______________
(1) Nuclear family (only parents and children) (2) Three-generation family (parents, children and grandparents) (3) Single parent family (4) Left-behind adolescents (both parents are not with you for a long time). (5) Weekend parents.

14. Are you the only child? _____________
(1)Yes (2) No

15. Your parents' marital status_____________
(1) Married and live together （2）Married but separated (3) Divorced (4) Widowed (5) Remarried

16. How about the quality of your father-mother relationship? _____________
(1) Very good (2) Relatively good (3) General (4) Relatively bad (5) Very bad

17. How about the quality of your mother-adolescent relationship? _____________
(1) Very good (2) Relatively good (3) General (4) Relatively bad (5) Very bad

18. How about the quality of your father-adolescent relationship? _____________
(1) Very good (2) Relatively good (3) General (4) Relatively bad (5) Very bad

19. How about your parental attitude toward your Internet-use? ____________
(1) Agree (2) Relatively agree (3) General (4) Relatively disagree (5) Strongly disagree

20. What’s your academic achievement level? ______________
(1) Very good (2) Relatively good (3) General (4) Relatively bad (5) Very bad

21. Does your family have computers at home? ___________
⑴ Yes (2) No If you choose (2), please skip to the 23th question.

22. Does your home computer have access to the Internet? _________
⑴ Yes (2) No

23. what's your usual place of using the internet? _____________
⑴ Internet bar (2) Home (3) Residences of relatives and classmates (4) School (5) If you never use
the Internet, you can end here and hand in this questionnaire to the investigator.

24. Usually, how many hours do you use internet during weekdays (from Monday to Friday) per week? ___ (1) Less than 2 hours (2) 2 to 4 hours (3) 4 to 6 hours (4) 6 to 8 hours (5) 8 hours or more

25. Usually, how many hours do you use the internet on weekends per week? _________
(1) Less than 2 hours (2) 2 to 4 hours (3) 4 to 6 hours (4) 6 to 8 hours (5) 8 hours or more

26. How many hours do you usually use the internet for a whole week? _________
(1)Less than 7 hours (2) 7 to 14 hours (3)14 to 21 hours (4) 21 to 28 hours (5)28 hours or more

27. What's your main purpose of using the Internet? ____________________
(1) Wandering aimlessly online (2) Only browsing news or email (3) Playing games (4) Real-time chatting (5) Academic learning

PART 2 DRM-52 Scale of Internet Use in Adolescents

1. Compared with the previous time, I spend more and more time on the internet ______________
(1) Completely disagree (2) Not so agree (3) General (4) Relative agree (5) Fully agree.

2. When off-line, I will imagine something related with the internet.____________________
(1) Completely disagree (2) Not so agree (3) General (4) Relative agree (5) Fully agree.

3. When distress, anxiety, irritability, depression, I go online, then I'll feel better____________
(1) Completely disagree (2) Not so agree (3) General (4) Relative agree (5) Fully agree.

4. I usually spend more time on-line than I intended._____________________
(1) Completely disagree (2) Not so agree (3) General (4) Relative agree (5) Fully agree.

5. I spend limited time on other entertainment activities to ensure enough time online_____
(1) Completely disagree (2) Not so agree (3) General (4) Relative agree (5) Fully agree.
6. My academic learning is not affected despite that I use the internet frequently.__________
(1) Completely disagree (2) Not so agree (3) General (4) Relative agree (5) Fully agree.

7. I'm unsatisfied if I spend the same amount of time online as before _________________
(1) Completely disagree (2) Not so agree (3) General (4) Relative agree (5) Fully agree.

8. I don't care if I need to cut down the amount of the online time____________________

(1) Completely disagree (2) Not so agree (3) General (4) Relative agree (5) Fully agree.

9. When I feel depressed, moody, or nervous when off-line, these negative emotions usually go away once I’m back on-line__________
(1) Completely disagree (2) Not so agree (3) General (4) Relative agree (5) Fully agree.

10. I think time flies when I’m online. I usually unwittingly spend more time online than I anticipated.________
(1) Completely disagree (2) Not so agree (3) General (4) Relative agree (5) Fully agree.

11. I spend less time on academic learning to ensure enough time online_____
(1) Completely disagree (2) Not so agree (3) General (4) Relative agree (5) Fully agree.

12. I don’t increase the frequency of using the internet._________________
(1) Completely disagree (2) Not so agree (3) General (4) Relative agree (5) Fully agree.

13. I know that the excessive use of the Internet will affect my academic learning, but I still go on using the Internet. _________
(1) Completely disagree (2) Not so agree (3) General (4) Relative agree (5) Fully agree.

14. I’ll feel distress, anxiety, irritability or depression if I can not go online for a period of time___________
(1) Completely disagree (2) Not so agree (3) General (4) Relative agree (5) Fully agree.

15. I never dream of anything related with the internet____________
(1) Completely disagree (2) Not so agree (3) General (4) Relative agree (5) Fully agree.

16. Going online always make me relaxed._________________
(1) Completely disagree (2) Not so agree (3) General (4) Relative agree (5) Fully agree.

17. When I’m on-line and plan to use the internet "just a few more minutes”, I can really do as I scheduled___
(1) Completely disagree (2) Not so agree (3) General (4) Relative agree (5) Fully agree.

18. I will do my best to get a faster Internet speed_____________
(1) Completely disagree (2) Not so agree (3) General (4) Relative agree (5) Fully agree.

19. I originally hope to spend very short time on-line, but later I find out that several hours have passed___
(1) Completely disagree (2) Not so agree (3) General (4) Relative agree (5) Fully agree.

20. When I want to cut down the amount of online time, I’m always able to do it.__________
(1) Completely disagree (2) Not so agree (3) General (4) Relative agree (5) Fully agree.

21. I would become restless if I go offline suddenly.______________
(1) Completely disagree (2) Not so agree (3) General (4) Relative agree (5) Fully agree.

22. When I feel fatigue, I will immediately stop using the internet to have a good rest__________
(1) Completely disagree (2) Not so agree (3) General (4) Relative agree (5) Fully agree.

23. The idea of going on-line always haunts me and makes me restless.___________
(1) Completely disagree (2) Not so agree (3) General (4) Relative agree (5) Fully agree.

24. I cannot help thinking of what is happening online.__________________
(1) Completely disagree (2) Not so agree (3) General (4) Relative agree (5) Fully agree.

25 Going online can not make me feel better when I feel bad.____________
(1) Completely disagree (2) Not so agree (3) General (4) Relative agree (5) Fully agree.

26. I suffer pain in the shoulder or other joint due to long periods of computer use, but I will continue using the internet in spite of the discomfort___________
(1) Completely disagree (2) Not so agree (3) General (4) Relative agree (5) Fully agree.

27. I can strictly obey the schedules of using the internet made by my parents or myself.__________
(1) Completely disagree (2) Not so agree (3) General (4) Relative agree (5) Fully agree.

28. Even when I feel very hungry, I will still use the internet until everything on the internet is done.______
(1) Completely disagree (2) Not so agree (3) General (4) Relative agree (5) Fully agree.

29. I have never purchased any items online for online games, such as point cards and game props____
(1) Completely disagree (2) Not so agree (3) General (4) Relative agree (5) Fully agree.

30. I am able to control the frequency of using the Internet by myself as originally planed__________
(1) Completely disagree (2) Not so agree (3) General (4) Relative agree (5) Fully agree.

31. I spend more and more time to stay indoor because of using the internet._____________
(1) Completely disagree (2) Not so agree (3) General (4) Relative agree (5) Fully agree.

32. When off-line, my fingers will casually or involuntarily make keyboard-typing action ____________
(1) Completely disagree (2) Not so agree (3) General (4) Relative agree (5) Fully agree.

33. I choose to spend more time on-line instead of going out with others._____________
(1) Completely disagree (2) Not so agree (3) General (4) Relative agree (5) Fully agree.

34. I completely forget about the time when I’m on-line.__________________
(1) Completely disagree (2) Not so agree (3) General (4) Relative agree (5) Fully agree.

35. I often communicate the feelings of being on-line with others._______________
(1) Completely disagree (2) Not so agree (3) General (4) Relative agree (5) Fully agree.

36. I never quarrel with my parents, teachers or other elders because of their disagreement toward my internet-use.______
(1) Completely disagree (2) Not so agree (3) General (4) Relative agree (5) Fully agree.

37. I can always find something to support me to use the internet.____________
(1) Completely disagree (2) Not so agree (3) General (4) Relative agree (5) Fully agree.

38. I have never been absent from school due to going online.______________
(1) Completely disagree (2) Not so agree (3) General (4) Relative agree (5) Fully agree.

39. I think it unnecessary to update my computer configuration in order to go online._____________
(1) Completely disagree (2) Not so agree (3) General (4) Relative agree (5) Fully agree.

40. My parents complain to me that I spend too much time online.__________________
(1) Completely disagree (2) Not so agree (3) General (4) Relative agree (5) Fully agree.

41. When I go off-line, I did not feel any discomfort.__________________
(1) Completely disagree (2) Not so agree (3) General (4) Relative agree (5) Fully agree.

42. I usually repeatedly go to the websites and forums I’m interested in, and repeatedly check my e-mails during one day._______________
(1) Completely disagree (2) Not so agree (3) General (4) Relative agree (5) Fully agree.

43. I usually don’t go on-line when I’m busy with academic learning._______________
(1) Completely disagree (2) Not so agree (3) General (4) Relative agree (5) Fully agree.

44. I prefer to form new relationships with on-line fellow users instead of making face-to-face friends in real life.__________
(1) Completely disagree (2) Not so agree (3) General (4) Relative agree (5) Fully agree.

45. I feel guilty when I spend much time online._____________________
(1) Completely disagree (2) Not so agree (3) General (4) Relative agree (5) Fully agree.

46. I prefer to use the internet when my parents are not at home, I feel more relaxed in such situation._____
(1) Completely disagree (2) Not so agree (3) General (4) Relative agree (5) Fully agree.

47. I prefer the excitement of the Internet to intimacy with my friends, sometime, I will miss meals or classes because of using the Internet.______________

(1) Completely disagree (2) Not so agree (3) General (4) Relative agree (5) Fully agree.

48. I'm not secretive when others ask me what I do on-line and how long I've been online._____________

(1) Completely disagree (2) Not so agree (3) General (4) Relative agree (5) Fully agree.

49. I will not have the slightest feeling of discomfort if I don't use the Internet for a period of time_____

(1) Completely disagree (2) Not so agree (3) General (4) Relative agree (5) Fully agree.

50. I hope to know what is happening online. __________________

(1) Completely disagree (2) Not so agree (3) General (4) Relative agree (5) Fully agree.

51. I find myself anticipating to go on-line even if I just go offline.______________

(1) Completely disagree (2) Not so agree (3) General (4) Relative agree (5) Fully agree.

52. I never lose sleep due to late-night log-ins. ________________

(1) Completely disagree (2) Not so agree (3) General (4) Relative agree (5) Fully agree.

上海市青少年因特网使用情况调查

所属区县______学校名称_______年级______　　填写日期____年___月___日

亲爱的同学：

我们想了解一下您网络使用的情况，为了保密，此问卷不记名，希望您不要有什么顾虑，回答您真实的情况和想法而不必考虑他人的评价。填写问卷时请注意问卷上的说明，用钢笔或圆珠笔在答案的题号上画“O”。答毕请检查一遍，不要漏项。占用您宝贵的时间。

谢谢合作！

一、一般情况：

1.您的性别？(　　)　（1）男　（2）女

2.您的出生年月_____年_____月___日

3.您是否寄宿(　　)　（1）是（2）否

4.您父亲的文化程度是：(　　)

⑴小学及以下　⑵初中　⑶高中(包括职高、技校、中专)　⑷大专及以上

5.您母亲的文化程度是：(　　)

⑴小学及以下　⑵初中　⑶高中(包括职高、技校、中专)　⑷大专及以上

6.您父亲的职业是：(　　)

⑴工人(制造、生产、运输人员) ⑵农民(农林牧渔业生产人员) ⑶商业从业人员 ⑷金融财务人员 (5)教师 (6)科技工作者 (7)行政干部 (8)医护人员 (9)待业人员 (10)其他(请注明)

7.您母亲的职业是：(　　)

⑴工人(制造、生产、运输人员) ⑵农民(农林牧渔业生产人员) ⑶商业从业人员 ⑷金融财务人员 (5)教师 (6)科技工作者 (7)行政干部 (8)医护人员 (9)待业人员 (10)其他(请注明)

8.您父母每月给您多少零用钱？(　　)

⑴100元以下　⑵100～299元　⑶300～599元　⑷600元以上

9.您自己月平均消费水平是：(　　)

⑴100元以下　⑵100～299元　⑶300～599元　⑷600元以上

10.您目前住的房子的性质是：(　　)　⑴家人购买的　⑵租赁的

11.您有自己独用的房间吗？(　　)　⑴有　⑵没有

12.您家里有汽车吗？(　　)　⑴有　⑵没有

13.您家的家庭类型　(　　)

⑴核心家庭（仅父母和子女）　⑵三代同堂　⑶单亲型　⑷父母一方长期不在身边　⑸仅周末和父母在一起

14.您是否独生子女　(　　)　⑴是　⑵不是

15.您父母婚姻状况(　　)　⑴在婚　⑵分居　⑶离婚　⑷丧偶　⑸再婚

16.您父母关系　(　　)　⑴.很好　⑵.较好　⑶.一般　⑷.较差　⑸.很差

17.您与父亲的关系如何？(　　)　⑴.很好　⑵.较好　⑶.一般　⑷.较差　⑸.很差

18.您与母亲的关系如何？(　　)　⑴.很好　⑵.较好　⑶.一般　⑷.较差　⑸.很差

19.父母对您上网的态度？(　　)

⑴.非常赞成　⑵.较赞成　⑶.无所谓　⑷.较反对　⑸.强烈反对

20. 您的学习成绩

⑴.很好　⑵.较好　⑶.一般　⑷.较差　⑸.很差

21.您家里有电脑吗？(　　　)

⑴有　⑵没有，　若选（2），跳至23题

22.您家里的电脑可以上网吗？(　　　)

⑴可以　　⑵不可以

23.您平时上网地点在(　　　)

⑴网吧　⑵家里　　　⑶亲戚朋友或同学家　⑷学校　　（5）从不上网，此次问卷调查结束

24.您周一至周五平均每日上网(　　　)

⑴≤2小时　⑵2~4小时　⑶4~6小时　⑷6~8小时　⑸8小时以上

25.您周六周日平均每日上网(　　　)

⑴≤2小时　⑵2~4小时　⑶4~6小时　⑷6~8小时　⑸8小时以上

26.您每周总上网时间(　　　)

⑴≤7小时　⑵7~14小时　⑶14~21小时　⑷21~28小时　⑸28小时以上

27.您上网主要是(　　　)

⑴无特别目的，随便看看　⑵仅新闻检索或单纯收发邮件　⑶游戏　　⑷在线聊天　⑸学习

二、DRM青少年因特网使用情况52量表

1.同以前相比，我上网的时间越来越长了。(　　　)

⑴.完全不符合　　　⑵.不太符合　⑶.一般　　　⑷.比较符合　　　⑸.完全符合

2.不上网的时候，我会幻想和上网有关的事情。(　　　)

⑴.完全不符合　　　⑵.不太符合　⑶.一般　　　⑷.比较符合　　　⑸.完全符合

3.当有苦恼、焦虑、烦躁、沮丧等不良感觉时，我就上网，这样我会感觉好一些。(　　　)

⑴.完全不符合　　　⑵.不太符合　⑶.一般　　　⑷.比较符合　　　⑸.完全符合

4.我上网的时间往往比计划的时间要长。(　　　)

⑴.完全不符合　　　⑵.不太符合　⑶.一般　　　⑷.比较符合　　　⑸.完全符合

5.为了上网，我会减少其他娱乐的时间。(　　　)

⑴.完全不符合　　　⑵.不太符合　⑶.一般　　　⑷.比较符合　　　⑸.完全符合

6.尽管经常上网，但我的学习成绩并没有下降。(　　　)

⑴.完全不符合　　　⑵.不太符合　⑶.一般　　　⑷.比较符合　　　⑸.完全符合

7.我上网的时间和以前相同，但心里已经感到不满足了。(　　　)

⑴.完全不符合　　　⑵.不太符合　⑶.一般　　　⑷.比较符合　　　⑸.完全符合

8.减少上网的时间对我而言无所谓。(　　　)

⑴.完全不符合　　　⑵.不太符合　⑶.一般　　　⑷.比较符合　　　⑸.完全符合

9.当情绪低落时，上网会使我变得心情舒畅。(　　　)

⑴.完全不符合　　　⑵.不太符合　⑶.一般　　　⑷.比较符合　　　⑸.完全符合

10.我觉得在网上时间过得很快，不知不觉就超过了自己预想的时间。(　　　)

⑴.完全不符合　　　⑵.不太符合　⑶.一般　　　⑷.比较符合　　　⑸.完全符合

11.因为上网，我减少了学习的时间。(　　　)

⑴.完全不符合　　　⑵.不太符合　⑶.一般　　　⑷.比较符合　　　⑸.完全符合

12.我上网的次数并没有增加。(　　　)

⑴.完全不符合　　　⑵.不太符合　⑶.一般　　　⑷.比较符合　　　⑸.完全符合

13.虽然知道上网会影响学习，但我还是继续上网。(　　)
⑴.完全不符合　⑵.不太符合　⑶.一般　⑷.比较符合　⑸.完全符合
14.如果有一段时间停止上网，我会感到坐立不安。(　　)
⑴.完全不符合　⑵.不太符合　⑶.一般　⑷.比较符合　⑸.完全符合
15.我从来没有梦到过关于网络的事情。(　　)
⑴.完全不符合　⑵.不太符合　⑶.一般　⑷.比较符合　⑸.完全符合
16.我发现上网是使我心情放松的好方法。(　　)
⑴.完全不符合　⑵.不太符合　⑶.一般　⑷.比较符合　⑸.完全符合
17.上网时，我发现自己会想"只要再过几分钟就下线"，结果真的就做到了。(　　)
⑴.完全不符合　⑵.不太符合　⑶.一般　⑷.比较符合　⑸.完全符合
18.我想方设法让自己的电脑上网速度更快。(　　)
⑴.完全不符合　⑵.不太符合　⑶.一般　⑷.比较符合　⑸.完全符合
19.我决定花较短的时间上网，但后来才发现好几个小时已经过去了。(　　)
⑴.完全不符合　⑵.不太符合　⑶.一般　⑷.比较符合　⑸.完全符合
20.当我想减少自己上网时间的时候，我总能做到。(　　)
⑴.完全不符合　⑵.不太符合　⑶.一般　⑷.比较符合　⑸.完全符合
21.突然停止上网，我就会变得烦躁不安。(　　)
⑴.完全不符合　⑵.不太符合　⑶.一般　⑷.比较符合　⑸.完全符合
22.当我在网上感到疲劳时，我会立刻下线休息。(　　)
⑴.完全不符合　⑵.不太符合　⑶.一般　⑷.比较符合　⑸.完全符合
23.想上网的念头一直困扰着我，使我烦躁不安，因此我会去上网。(　　)
⑴.完全不符合　⑵.不太符合　⑶.一般　⑷.比较符合　⑸.完全符合
24.我会情不自禁地想起网上发生的事情。(　　)
⑴.完全不符合　⑵.不太符合　⑶.一般　⑷.比较符合　⑸.完全符合
25.当我觉得心情烦躁时，即使上网也不会使我减轻这种感受。(　　)
⑴.完全不符合　⑵.不太符合　⑶.一般　⑷.比较符合　⑸.完全符合
26.由于长时间坐在电脑旁，我的肩或其他关节痛，但这并不影响我继续上网。((　　)
⑴.完全不符合　⑵.不太符合　⑶.一般　⑷.比较符合　⑸.完全符合
27.我能严格遵守父母或者自己规定的上网时间。(　　)
⑴.完全不符合　⑵.不太符合　⑶.一般　⑷.比较符合　⑸.完全符合
28.虽然很饿了，但我还是会把网上的事情处理完后再吃饭。 (　　)
⑴.完全不符合　⑵.不太符合　⑶.一般　⑷.比较符合　⑸.完全符合
29.我从来没有参与过网上虚拟物品的交易（包括点卡、游戏道具、账号等）。(　　)
⑴.完全不符合　⑵.不太符合　⑶.一般　⑷.比较符合　⑸.完全符合
30.我能够有计划地控制自己的上网次数。(　　)
⑴.完全不符合　⑵.不太符合　⑶.一般　⑷.比较符合　⑸.完全符合
31.因为上网，我在室内呆的时间越来越长了。(　　)
⑴.完全不符合　⑵.不太符合　⑶.一般　⑷.比较符合　⑸.完全符合
32.不上网的时候，我的手指会随意或不随意地做出敲击键盘的动作(　　)
⑴.完全不符合　⑵.不太符合　⑶.一般　⑷.比较符合　⑸.完全符合

33.我减少了拜访朋友的时间而宁愿在网上逗留。(　　)
⑴.完全不符合　⑵.不太符合　⑶.一般　⑷.比较符合　⑸.完全符合
34.上网后我就忘记了时间。(　　)
⑴.完全不符合　⑵.不太符合　⑶.一般　⑷.比较符合　⑸.完全符合
35.我常常和周围人交流使用网络的心得。(　　)
⑴.完全不符合　⑵.不太符合　⑶.一般　⑷.比较符合　⑸.完全符合
36.我从来没有因为上网的原因而与父母、老师等长辈产生过争执。(　　)
⑴.完全不符合　⑵.不太符合　⑶.一般　⑷.比较符合　⑸.完全符合
37.我常常能找出一些理由使我更多地使用网络。(　　)
⑴.完全不符合　⑵.不太符合　⑶.一般　⑷.比较符合　⑸.完全符合
38.我从没有因为上网而缺过课。(　　)
⑴.完全不符合　⑵.不太符合　⑶.一般　⑷.比较符合　⑸.完全符合
39.我觉得没有必要去为使用网络而特意更新电脑配置。(　　)
⑴.完全不符合　⑵.不太符合　⑶.一般　⑷.比较符合　⑸.完全符合
40.家人抱怨我花了太多的时间上网。(　　)
⑴.完全不符合　⑵.不太符合　⑶.一般　⑷.比较符合　⑸.完全符合
41.当我从网上离开时，我并没有不舒服的感觉。(　　)
⑴.完全不符合　⑵.不太符合　⑶.一般　⑷.比较符合　⑸.完全符合
42.一天中我常会多次查看自己感兴趣的网站、论坛以及自己的电子邮箱。(　　)
⑴.完全不符合　⑵.不太符合　⑶.一般　⑷.比较符合　⑸.完全符合
43.学习很忙时我就不会上网了。(　　)
⑴.完全不符合　⑵.不太符合　⑶.一般　⑷.比较符合　⑸.完全符合
44.比起面对面地与人交流，我更愿意在网上与人交流。(　　)
⑴.完全不符合　⑵.不太符合　⑶.一般　⑷.比较符合　⑸.完全符合
45.我对自己上网所花的时间感到内疚。(　　)
⑴.完全不符合　⑵.不太符合　⑶.一般　⑷.比较符合　⑸.完全符合
46.我会趁家人不在家时上网，这样我感觉比较轻松。(　　)
⑴.完全不符合　⑵.不太符合　⑶.一般　⑷.比较符合　⑸.完全符合
47.我会因上网错过吃饭、上课或与同学在一起的时间。(　　)
⑴.完全不符合　⑵.不太符合　⑶.一般　⑷.比较符合　⑸.完全符合
48.我没有向任何人隐瞒过我上网的实际情况(包括在线时间、上网次数等)。(　　)
⑴.完全不符合　⑵.不太符合　⑶.一般　⑷.比较符合　⑸.完全符合
49.即使减少或者停止上网一段时间，我也不会因此有丝毫不适的感觉。（　　）
⑴.完全不符合　⑵.不太符合　⑶.一般　⑷.比较符合　⑸.完全符合
50.不上网的时候，我很想知道网上发生的事情。(　　)
⑴.完全不符合　⑵.不太符合　⑶.一般　⑷.比较符合　⑸.完全符合
51.下线几个小时后我就又想上网了，其实才过了很短的时间。(　　)
⑴.完全不符合　⑵.不太符合　⑶.一般　⑷.比较符合　⑸.完全符合
52.我从来不会通宵上网。(　　)
⑴.完全不符合　⑵.不太符合　⑶.一般　⑷.比较符合　⑸.完全符合
